# Optical Response of PDMS Surface Diffraction Gratings under Exposure to Volatile Organic Compounds

**DOI:** 10.1021/acsaom.4c00138

**Published:** 2024-05-28

**Authors:** Aleksandra Hernik, Faolan Radford McGovern, Izabela Naydenova

**Affiliations:** Centre for Industrial & Engineering Optics, School of Physics, Clinical & Optometric Sciences, Technological University Dublin, D07 ADY7 Dublin, Ireland

**Keywords:** holographic sensors, volatile organic compounds, PDMS grating, surface relief gratings, holography

## Abstract

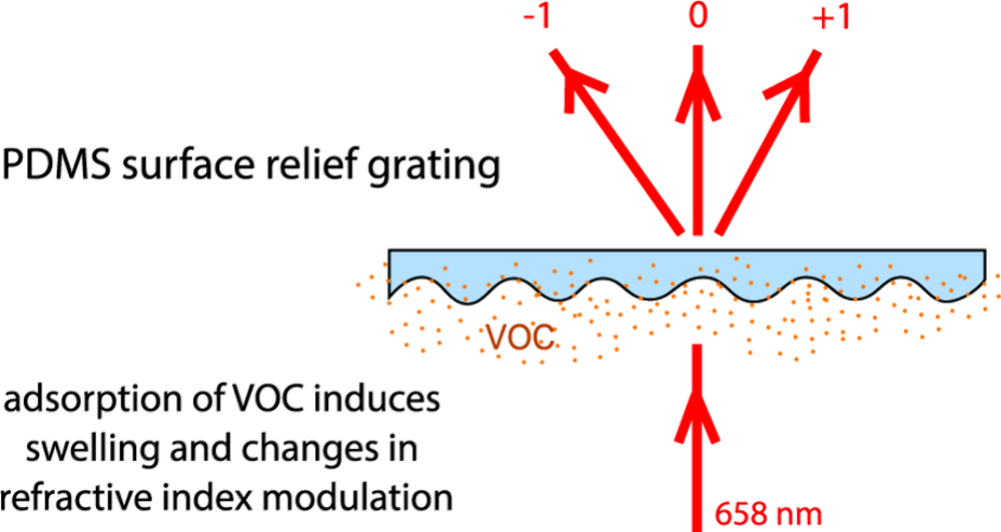

Monitoring volatile organic compounds (VOCs) in indoor air is significantly gaining importance due to their adverse effects on human health. Among the diverse detection methods is optical sensing, which employs materials sensitive to the presence of gases in the environment. In this work, we investigate polydimethylsiloxane (PDMS), one of the materials utilized for gas sensing, in a novel transducer: a surface relief diffraction grating. Upon adsorption of the volatile analyte, the PDMS grating swells, and its refractive index changes; both effects lead to increased diffraction efficiency in the first diffraction order. Hence, the possibility of VOC detection emerges from the measurement of the optical power transmitted or diffracted by the grating. Here, we investigated responses of PDMS gratings with varying surface profile properties upon exposure to VOCs with different polarities, i.e., ethanol, *n*-butanol, toluene, chloroform, and *m*-xylene, and compared their response in the context of the Hansen theory of solubility. We also studied the response of the grating with a 530 nm deep surface profile to different concentrations of *m*-xylene, showing a sensitivity and limit of detection of 0.017 μW/ppm and 186 ppm, respectively. Structures in the PDMS were obtained as copies of sinusoidal surface gratings fabricated holographically in acrylamide photopolymer and revealed good sensing repeatability, reversibility, and a fast response time. The proposed sensing technique can be directly adopted as a simple method for VOC detection or can be further improved by implementing a functional coating to significantly enhance the sensitivity and selectivity of the device.

## Introduction

Volatile organic compounds (VOCs) are air pollutants that are able to cause severe damage to human health, especially after long-term exposure. Sources of VOCs are highly diverse, which makes them almost ineradicable from an indoor environment. Among the common sources are paints, cleaning agents and aerosols, personal care products, building materials, and furnishing.^1^ Limits of concentration of certain gases are regulated by environmental protection agencies.^2−4^ Exceeding those limits in indoor air may cause serious health effects, including eye, nose, and throat irritation, respiratory issues, headaches and nausea, organ damage, and even cancer.

To effectively control the quantity of VOCs in indoor air, sensitive and reliable detectors must be introduced. The most widely used sensing technologies include gas chromatography,^5^ absorption spectroscopy,^6,7^ photoionization detection,^8^ fiber-^9,10^ and photonic crystal-based^11^ sensing, MEMS systems,^12^ and resistive-based metal oxide^13,14^ and non-oxide^15^ sensing. In the context of optical sensors, such as those based on photonic crystals or optical fibers, the selection of a suitable material holds the key importance for detecting the presence of VOCs within the ambient environment. This chosen material, after absorption or other chemical interaction with the analyte, will most often react by swelling, or a change in the refractive index will occur. As a result, these interactions lead to changes in the wavelength and/or intensity of light either reflected or transmitted through the sensor, thus indicating analyte presence.^16^ Among the frequently utilized transducers’ materials are porous silicon,^17,18^ metal–organic frameworks,^19−21^ and polydimethylsiloxane (PDMS).^22−27^

PDMS, a type of silicone elastomer, has desirable characteristics for an optical sensing medium and applications in remote devices due to its transparency, flexibility, biocompatibility, mechanical stability, and humidity resistance. It was successfully implemented as a distributed Bragg reflector,^22^ a composite of colloidal crystal,^23^ a coating for fiber Bragg gratings,^24−26^ and as a volume holographic grating medium,^27^ all of them designed for VOC sensing. The response of PDMS to various vapors may be attributed to two mechanisms upon absorption, i.e., swelling or a change in the refractive index, as described by Saunders et al.^28^ With the use of an interferometric refractometer and by implementing Fourier transform-based data analysis, the researchers showed simultaneous measurements of refractive index and film thickness.^29^ Results proved that measuring those two parameters is sufficient to calculate the gas concentration that permeated into the polymer film. They also confirmed a much stronger response of PDMS to nonpolar solvents like xylene, toluene, and benzene and low sensitivity to polar solvents, namely acetone, methanol, and isopropanol.^28^ Swelling of the material upon interaction with a solvent may be predicted with the help of the Hansen theory of solubility.^30^ According to this theory, three parameters can be linked to the material based on energy coming from hydrogen bonding, dispersion, and intermolecular interactions. If the parameters of PDMS are similar to those of the investigated solvent, then the analyte will more likely dissolve in PDMS and cause swelling. A good agreement between Hansen theory and the swelling of PDMS in solvent vapors with different pressures was demonstrated by Rumens et al.^31^

An example of structures whose interaction with an electromagnetic wave is predominantly based on spatial dimensions and refractive index are two-dimensional diffraction gratings. Some of them were already proposed as sensing devices for VOCs,^32−36^ yet this area remains relatively unexplored. A distinctive subgroup within this category comprises surface structures fabricated using holographic methods.^37,38^ These gratings, although possessing the potential to offer exceptionally precise dimensions of sinusoidal surface relief profiles, have rarely been employed as gas sensors so far,^37,39−41^ even though an adequate theoretical study has been proposed.^42^

Here, we present the use of a pure PDMS surface relief grating to detect VOCs in ambient air. The novelty of the present work is in the selected type of optical transducer (its design, fabrication, and characterization) and its application in VOC detection. The main advantage of such a transducer is the inherent high sensitivity and potential flexibility in functionalization by surface coatings for achieving high selectivity. To the best of our knowledge, we demonstrate the successful detection of VOCs utilizing diffraction from PDMS surface relief gratings for the first time. Gratings were prepared as copies of sinusoidal surface relief structures inscribed in acrylamide photopolymer using the holographic method. We investigated the dependence on the spatial profile of the grating to the response signal when exposed to vapor analytes with different polarities and the expected swelling ratio, according to the Hansen theory of solubility. Therefore, the response of PDMS to five VOCs was studied, namely *m*-xylene, *n*-butanol (or 1-butanol), toluene, ethanol, and chloroform.

## Theory

### VOC-Induced Polymer Swelling

Polymers and in particular PDMS have been demonstrated to swell in the presence of certain VOCs.^43^ This effect has been utilized in many VOC sensors using several detection methods, such as fiber interferometric and capacitive type sensors.^23,44−47^ The extent to which a certain VOC will induce swelling in a polymer can be examined using solubility parameters.

#### Hansen Solubility Parameters

A common method of determining the compatibility of polymers and solvents is using the Hansen solubility parameters (HSPs). The method is based on three solubility parameters: the dispersion (δ_d_), polar (δ_p_), and hydrogen bonding (δ_H_) components.^30^ The basis of the model is that “like dissolves like”, meaning polymers and solvents with similar HSP values should be compatible.^48^ While the HSP values are directly related to solubility, there is a correlation with swelling effects.^30^ Nielsen and Hansen^49^ have examined the relationship between elastomer swelling and HSPs, finding that it is possible to predict elastomer swelling in contact with solvents. For ethylene propylene diene monomer rubber elastomers, it was found that solvents with similar HSPs to the elastomer induced higher degrees of swelling than when a large difference in the HSPs was present.^49^ The parameter *R*_a_, characterizing the interaction of a solvent (1) and polymer (2) in terms of HSPs, is given by^30,48,50^
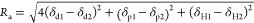
1To determine if a solvent is compatible with a polymer, the relative energy difference (RED) is used,^48^ given as
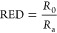
2where *R*_0_ is the Hansen interaction sphere radius for the polymer.^51^ For a good solvent, RED < 1, while RED > 1 indicates poor compatibility.^30^ HSPs can be plotted on a three-dimensional (3D) plot, and should the solvents fall within the Hansen sphere for the polymer (radius *R*_0_), they are considered to be a good solvent. The use of HSPs has been seen to evaluate several PDMS-based sensors.^24,31^

#### PDMS VOC Solvency

**Figure 1:**
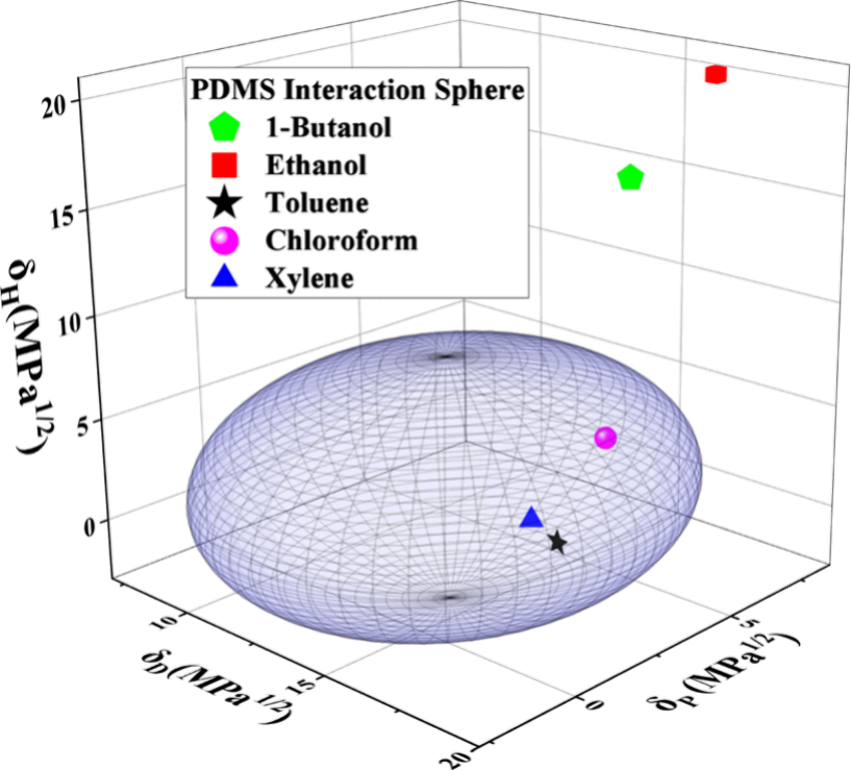
Hansen interaction sphere for PDMS with the VOCs examined in this study.

PDMS is an optically clear, hydrophobic, biocompatible elastomer with numerous sensing and engineering applications.^52^ The compatibility of PDMS with a number of organic solvents has been previously investigated.^53^ Molecularly, the PDMS structure is helical, composed of a curved Si–O–Si chain with a layer of nonpolar methyl −CH_3_ groups around the outside of the chain.^54^ Thus, the most swelling of PDMS occurs in the presence of nonpolar solvents such as pentane and xylenes, where the dispersion forces are the primary contributor to the swelling parameter; polar solvents such as water and ethylene glycol causing less swelling.^53^ The swelling effects of PDMS in the presence of VOCs have been used in several sensors.^31,55,56^ In addition to swelling, VOC interaction with PDMS also results in a change in the refractive index, which is also used as a sensing effect.^57,58^ The dissolution and diffusion characteristics of polysiloxane materials have also been investigated in terms of improving selectivity.^59^ A number of works involving PDMS used as a VOC sensor have attempted to predict the response of the device using HSPs. In terms of HSPs, PDMS can be considered a special case^60^ with several different HSPs reported.^61−66^ In this study, Sylgard 184 PDMS is used at a mixing ratio of 10:1, as is the case in the work presented by Ollé et al.,^62^ where HSP values of δ_d_ = 15.9 MPa^1/2^, δ_p_ = 0.1 MPa^1/2^, and δ_H_ = 4.7 MPa^1/2^ are assumed. The interaction radius *R*_0_ for PDMS is reported as both 5.7 and 5.6 MPa^1/2^.^63,66^ Using an approach similar to Kanawade et al., we took a value of *R*_0_ = 5.6 MPa^1/2^ to calculate the RED between PDMS and the VOCs examined in this study. Furthermore, the data in Table 1 can be graphically represented using the Hansen sphere, as illustrated in Figure 1.

**Table 1:** HSP Values for VOCs Examined in This Study^30^ and Relative RED Values for PDMS

VOC	δ_d_	δ_p_	δ_H_	*R*_a_	RED
ethanol	15.8	8.8	19.4	17.12	3.10
1-butanol	16.0	5.7	15.8	12.43	2.22
toluene	18.0	1.4	2.0	5.26	0.92
chloroform	17.8	3.1	5.7	4.94	0.88
xylene	17.6	1.0	3.1	3.86	0.69

Based on the data in Table 1, toluene, chloroform, and xylene have RED values <1, indicating they are good solvents for PDMS. As seen in Figure 1, they fall within the PDMS interaction sphere. As such, it is expected that these VOCs will result in the largest sensor response. Conversely, ethanol and 1-butanol fall outside the interaction sphere with RED values >1, indicating that they are poor solvents for PDMS; consequently, the sensor is not expected to detect these analytes with a significant response.

### Surface Diffraction Grating as a Sensor

A holographic surface grating is formed as a sinusoidal, periodical modulation of surface thickness. Being optical diffractive sensors, surface gratings operate via changes in refractive index or due to swelling upon exposure to the analyte. Their diffraction efficiency, i.e., the ratio of the intensity *I* of light diffracted in selected order *m* to the intensity of the incident light, according to the Raman–Nath theory^67^ follows eq 3:
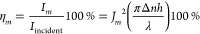
3where Δ*n* is the refractive index modulation, *h* is the surface relief height, and λ is the wavelength of the incident light. *J*_*m*_^2^ refers to the squared Bessel function of the first kind, indicating a nonlinear response of the sensor over the large range of phase modulation changes. Even though swelling may also influence the grating period, the diffraction efficiency shows no dependence on spatial frequency. Hence, after the adsorption of vapor molecules, a change in Δ*n* and *h* may occur, both leading to a variation in the optical powers of beams propagating through the grating. In fact, even without adsorption, the mere changes in the refractive index of the surrounding air, contributing to Δ*n*, can lead to deviations in the intensity of diffracted light. Leveraging the information mentioned above, detection may be performed by measuring alterations in the intensity of light redirected into selected diffraction order. Conveniently, the first order is chosen for that purpose due to the higher diffraction efficiency and lower angle of diffraction than those in subsequent orders and because of the usually more precise readout compared to that in the zeroth order, which can be sensitive to other intensity losses not related to diffraction effects.

**Figure 2:**
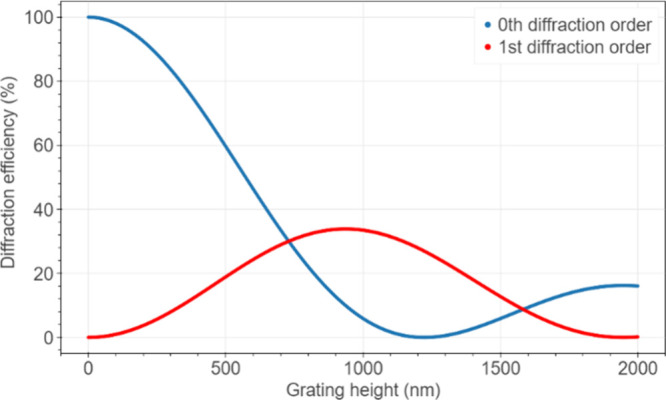
Dependence of diffraction efficiency in the zeroth and first diffraction orders on height of the sinusoidal PDMS surface grating for refractive index modulation Δ*n* = 0.41 and wavelength of incident light λ = 658 nm. Diffraction efficiency is independent of the grating’s spatial frequency.

The initial diffraction efficiency for gratings inscribed in the selected material and for a chosen wavelength will differ depending on the surface profile height, as depicted in Figure 2. Here, we assumed the refractive index of pure PDMS as *n* = 1.41.^68^ Therefore Δ*n* = 1.41 −1 = 0.41 is the difference between the refractive indices of the elastomer and the surrounding air. In the region of 120–530 nm of depths investigated in this work, the initial diffraction efficiency in the first order always tends to increase when the grating height increases. Simultaneously, the optical power diffracted into the zeroth order is expected to decrease.

**Figure 3:**
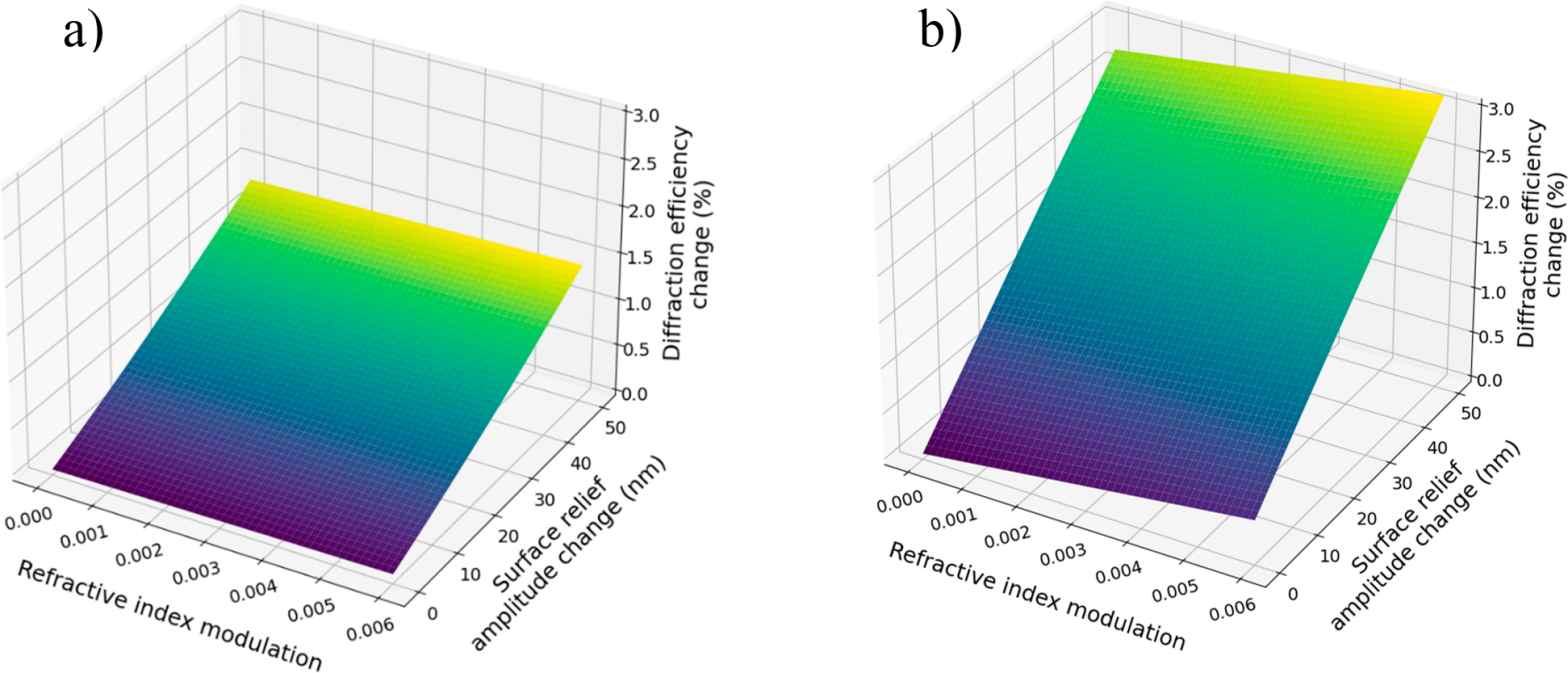
Change of diffraction efficiency upon modulation of refractive index and PDMS swelling for gratings with an initial depth of (a) 120 and (b) 530 nm. Surface relief amplitude change is due to swelling of the material.

The sensitivity of the sensor can be evaluated by taking the derivative ∂η/∂Δ*n* for refractive index modulation changes and ∂η/∂*h* for changes induced by polymer swelling. Figure 3 demonstrates the theoretical changes in diffraction efficiency calculated numerically when Δ*n* and *h* are growing due to exposure to a volatile analyte. The maximum changes of Δ*n* and *h*, i.e., 0.006 and 50 nm, respectively, were selected considering the results demonstrated by Saunders et al. for toluene.^28^ Gratings with different initial depths were compared, showing a much higher diffraction efficiency change for the deeper grating upon the same changes caused by the VOC, meaning that a higher concentration of gas is required to interact with the grating with a lower profile to provide the same signal as in the grating with a deeper profile. The swelling effect may be stipulated with the Hansen theory of solubility, as described previously. To predict changes in the refractive index of PDMS caused by the analytes, knowledge of the refractive indices of the analytes in their liquid form is very useful. Table 2 provides information on the refractive index of PDMS^68^ and the refractive indices of the VOCs studied in this paper.^69^ For comparative purposes, we refer to the refractive index of PDMS^68^ and the refractive indices of the VOCs studied in this paper.^69^ For comparative purposes, we refer to the VOCs in their liquid state.

**Table 2:** Refractive Indices of Selected VOCs^69^ (at 658 nm, in Liquid State) and PDMS^68^ (at 589 nm)

	toluene	*m*-xylene	chloroform	PDMS	*n*-butanol	ethanol
refractive index	1.49	1.49	1.44	1.41	1.40	1.36

According to Saunders et al.,^28^ two distinct mechanisms of the absorption of gas molecules into siloxane polymers may be present: (1) filling the “voids” in the polymer (thus, the volume is not changing) or (2) swelling of the polymer by a volume of adsorbed analyte.

In the first scenario, the refractive index will always increase after absorption. In the latter case, the refractive index may increase or decrease, depending on the refractive index of the compounds permeating into the material. A refractive index decrease is expected in the case of strong swelling, which almost always leads to a strong volume change, resulting in the reduction of the material’s density and thus decreasing the refractive index. Saunders et al. presented a small PDMS refractive index reduction upon exposure to acetone, methanol, and isopropanol.^28^Table 2 shows that within the VOCs investigated in this study, only *n*-butanol and ethanol may contribute to the decrease of the refractive index of PDMS. In the light of other mechanisms, all tending to increase the refractive index, this behavior may slightly weaken the sensing response to these two components. On the contrary, the strongest influence on the diffraction efficiency is expected to come from interactions with *m*-xylene and toluene.

## Experimental Methods

### Materials

A Sylgard 184 silicone elastomer kit, i.e., base and curing agent, was obtained from VWR Chemicals. Vapors of ethanol (ABS, 99%), toluene (HPLC grade), chloroform (reagent grade, Fisher Scientific), 1-butanol (ACS, 99.4%+, Fisher Scientific), and *m*-xylene (99%, Fisher Scientific) were used for sample testing without further modification.

### Fabrication of PDMS Surface Relief Diffraction Gratings

Master gratings with sinusoidal shapes, of which PDMS copies were prepared, had been fabricated holographically in acrylamide photopolymer following a previously described procedure.^70,71^ Briefly, a photopolymer mixture containing Methylene Blue as a sensitizer was poured on microscopic glass slides and left steadily to dry for 6 h. Then, samples were irradiated with two 660 nm laser beams of a total intensity of 1.1 mW/cm^2^ for 60 s (Cobolt Flamenco), creating an interference pattern consisting of dark and bright regions on the samples’ surface. Due to the polymerization-driven diffusion in the material, surface relief gratings were formed. To further polymerize any remaining monomer, samples were bleached with an ultraviolet (UV) light source (Mega Electronics, model 5503-11, 2.5 mW/cm^2^) for 30 min. Finally, gratings were thermally treated (Memmert model UNB 100) by gradually increasing the temperature from 70 to 220 °C at a rate of 1 °C/min. Once the samples reached 220 °C, they were left at this temperature for another 10 min in the oven. By changing the angle between two interfering beams in the optical setup, gratings with different spatial frequencies and, thus, different depths were obtained.

Polydimethylsiloxane was prepared by thoroughly mixing the base and curing agent of the Sylgard 184 silicone elastomer with a 10:1 ratio. A PDMS mixture was then poured on the master gratings placed in a mold and left steadily for 1 h for the material to enter uniformly into the surface profile of the grating. Then, PDMS films were cured at 60 °C for 1 h, peeled off from the master grating, and placed on a clean glass slide, with the plain side of the PDMS layer attached to the glass surface. Investigated PDMS layers had a 0.75 ± 0.25 mm thickness.

### Setup for VOC Exposure

**Figure 4:**
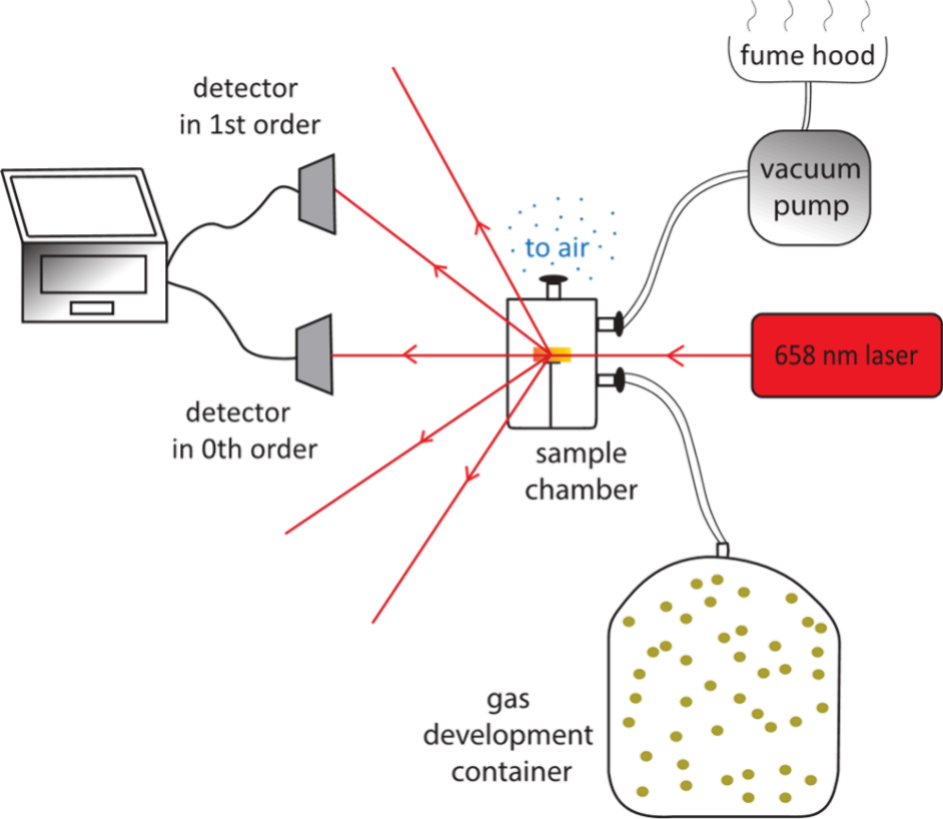
Setup for VOC exposure. The investigated grating is placed in a small sample chamber, connected to the gas development container with the analyte, vacuum pump, and ambient air. The optical power from the 658 nm laser, diffracted into the zeroth and first orders, is collected with photodetectors.

The PDMS gratings were exposed to vapors using a gas test setup, previously described in ref (72). It consisted of a small glass chamber of a cylindric shape connected to a gas development container, where VOCs in liquid state were injected to evaporate, as shown in Figure 4. During the first part of the experiment, 0.5 mL of the selected analyte was dispensed into the container and allowed to vaporize (the relative volumes of the gas exposure unit and the gas development container are 1:9), ensuring a significant gas concentration circulating around the sample. Exact concentrations, together with the calculation method, are provided in the Supporting Information. In the second experimental part, to determine sensor response upon varying concentrations of *m*-xylene, different amounts of liquid were injected to obtain expected concentrations of gas in the container. The chamber with the sample also had two additional connections: one to the vacuum pump and another enabling an opening to the air in the ambient room environment. A continuous wave laser diode operating at 658 nm was placed opposite to the sample, in front of the chamber; the probe beam was incident normally to the glass cylinder surface, and the sample was placed at the center of the cylinder. The laser was chosen based on its stable intensity output and sufficient power of 31.6 mW. The probe beam was adjusted to be incident on the sample at a location along the axis of the cylinder. The diffraction pattern was visible in transmission on the opposite side of the sample. The hologram was placed in the center of the cylinder in order to ensure that the emerging diffraction beams were also orthogonal to the cylinder surface and thus decrease any losses in intensity due to reflection from the surface of the sample chamber. The probe beam power, measured after its propagation through both glass boundaries of the empty cylinder, was measured to decrease to 25.28 ± 0.89 mW due to scattering losses. Two silicon photodetectors were placed into the zeroth and first diffraction orders to measure the optical power of light that propagated through the sample and diffracted, respectively.

Measurements were taken in few-minute cycles, first evacuating the chamber with the sample inside and then opening the valve to ambient air or to the gas development container with vapor. Due to the underpressure created by evacuating, gas filled the chamber immediately after opening the connection. Evacuation itself influences the PDMS grating with the change in diffraction efficiency; hence, the sample’s response to air is perceived as a base level diffracted signal. The sensor response to the analyte was determined as the difference in the two signals: the measured optical power when the chamber was filled with air and the optical power when the chamber was filled with the gaseous analyte.

## Results and Discussion

**Figure 5:**
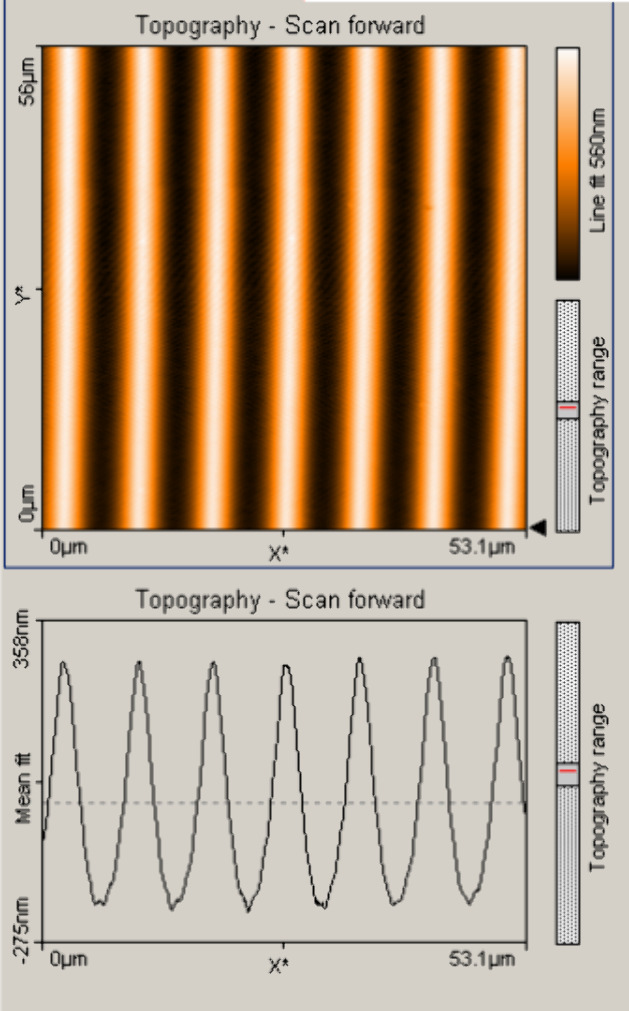
Atomic force microscopy image of the grating with a 490 nm height and a 8 μm period. The grating is a PDMS copy of the sinusoidal surface relief grating fabricated in acrylamide photopolymer.

Gratings with different periods Λ, ranging from 2 to 8 μm, and surface relief depths *d* in the range 120–530 nm were selected for investigation. Surface profiles of every grating were characterized with an atomic force microscope (AFM). An example of the surface profile can be seen in Figure 5. AFM images of the remaining four gratings are shown in Figure S1 in the Supporting Information. For lower spatial frequencies (higher grating periods), the obtained surface depths were higher, which emerge from the properties of the photopolymer during holographic irradiation (a higher period of interference pattern enables the fabrication of deeper surface gratings).^70,71^Table 3 summarizes the profile characteristics of the fabricated PDMS gratings and their theoretical diffraction efficiencies in the first and zeroth orders. Among them, two gratings with the same period of 8 μm but different depths were fabricated. Gratings with higher depths reach higher diffraction efficiencies, as calculated by applying Raman–Nath theory for thin sinusoidal diffraction gratings with characteristics (refractive index, and surface relief amplitudes) similar to those of the fabricated gratings and probed at the same wavelength, as depicted in Figure 2. The agreement between the theoretically predicted and experimentally measured diffraction efficiencies is very good. The variation in the measured experimentally diffraction efficiency can be explained by light scattering losses, slightly varying relief depths along the grating surface, and possible variations in the refractive index of the prepared PDMS.^68^

**Table 3:** Surface Profiles of the Examined Gratings, Diffraction Efficiencies Calculated with Raman–Nath Theory and Associated with Surface Heights, and Experimentally Measured Diffraction Efficiencies in the First Diffraction Order^a^

period (μm)	height (nm)	theory η_0_ (%)	theory η_1_ (%)	experiment η_1_ (%)
2.1	120	97.2	1.4	0.74 ± 0.04
7.2	210	91.7	4.1	3.6 ± 0.6
7.9	400	72.4	13.2	14.4 ± 3.3
8.0	490	61.0	18.3	17.3 ± 1.3
8.0	530	55.7	20.6	18.7 ± 1.6

a Grating periods and heights were determined by AFM measurements.

Further, we describe the results obtained during the exposure cycles of PDMS gratings with different surface relief heights to VOCs with high concentrations (>10 000 ppm). Every sample was first exposed to a vacuum and then exposed to ambient air to determine the background power level in the zeroth and first orders of diffraction, i.e., in an analyte-free environment. This procedure was repeated several times to ensure the reversibility of the sensor before exposure to the analyte. Here, we focus on a change of power when the analyte is present in relation to the value read when the vacuum pump was running at the beginning of each measurement for the convenience of analysis. Thus, the total optical power change due to exposure to a VOC may be interpreted as the difference between the value obtained when the chamber with the sample was opened to the ambient air and the value obtained when the gas from the development container (i.e., mixture of air and evaporated analyte) entered the chamber.

**Figure 6:**
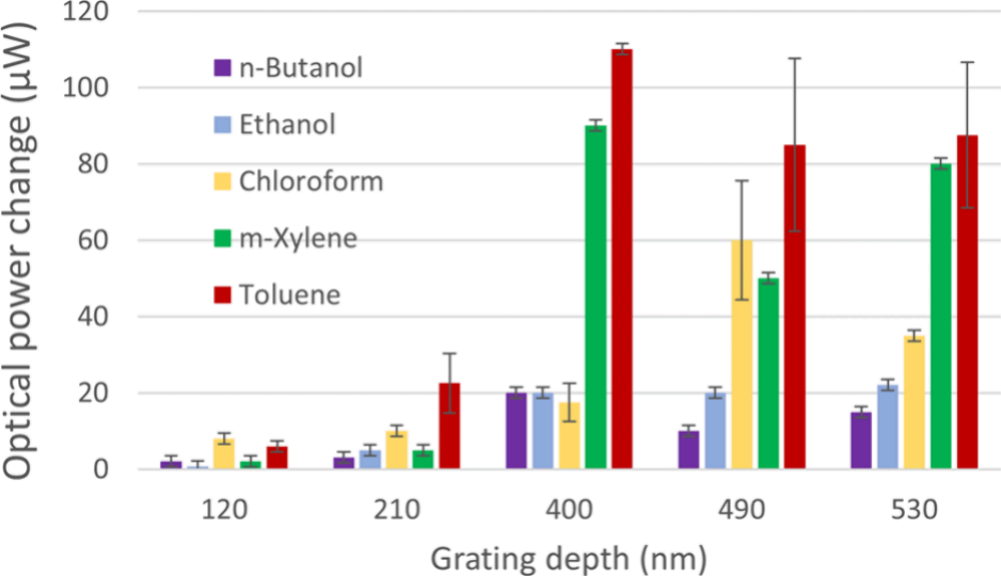
Optical power change in the first order of diffraction for gratings with different depths of the surface profile after exposure to VOCs. The change is determined as the difference in power measured when the sample, after exposure to vacuum, was exposed to ambient air and to the analyte. Error bars were estimated based on the mechanical stability of the sensor, and when more than one measurement cycle was performed, the standard deviation from all responses was added.

For each of the investigated VOCs (*m*-xylene, toluene, chloroform, *n*-butanol, and ethanol), a series of measurements was performed for each PDMS grating. The gathered results (for the first order of diffraction) are presented in Figure 6. As the final responses, the maximum measured power changes from the baseline value, defined as the power level during the last exposure to air, were taken. Normally, two or three experiments involving multiple cycles were conducted, providing an average response with the deviations expressed with error bars. Comparing the responses depending on the grating surface profile, it is clear that the three gratings with the deepest investigated surface reliefs are significantly more sensitive to VOCs than the gratings with depths of 120 and 210 nm, as predicted using Raman–Nath theory (Figure 3). The highest response was measured for toluene exposure in almost every case, with an optical power change up to 110 μW and a corresponding diffraction efficiency change of 0.44 ± 0.02%. The VOC causing the second largest response was *m*-xylene, with responses reaching 90 μW. Still relatively high optical power differences up to 70 μW were observed when chloroform permeated the samples. Responses to *n*-butanol and ethanol did not exceed 20 μW, being slightly higher for *n*-butanol, despite the fact that the concentration was the highest for these two analytes (see Table S1). The above results are in line with the Hansen theory of solubility, as the three nonpolar VOCs showed much higher responses than the polar analytes.

Nevertheless, it can be observed that the obtained results do not match the Hansen theory fully, as according to this theory, the response to *m*-xylene should be prevailing, while the lowest response is expected for exposure to ethanol. Most likely, the observed deviations from the Hansen theory are due to the variations of concentrations of VOCs generated, as the same amount of liquid (0.5 mL) was deposited in the development chamber for each of the gases under study (see Table S1). To a lesser extent, the difference in the response may also be related to the second dominant effect, namely a change in the refractive index. Furthermore, other chemical interactions, which cannot be predicted by the Hansen theory, may take place and contribute to the swelling. It is worth noting that there are other experimental reports where researchers have obtained a stronger response of PDMS when exposed to toluene than to *m*-xylene.^23,28^

**Figure 7:**
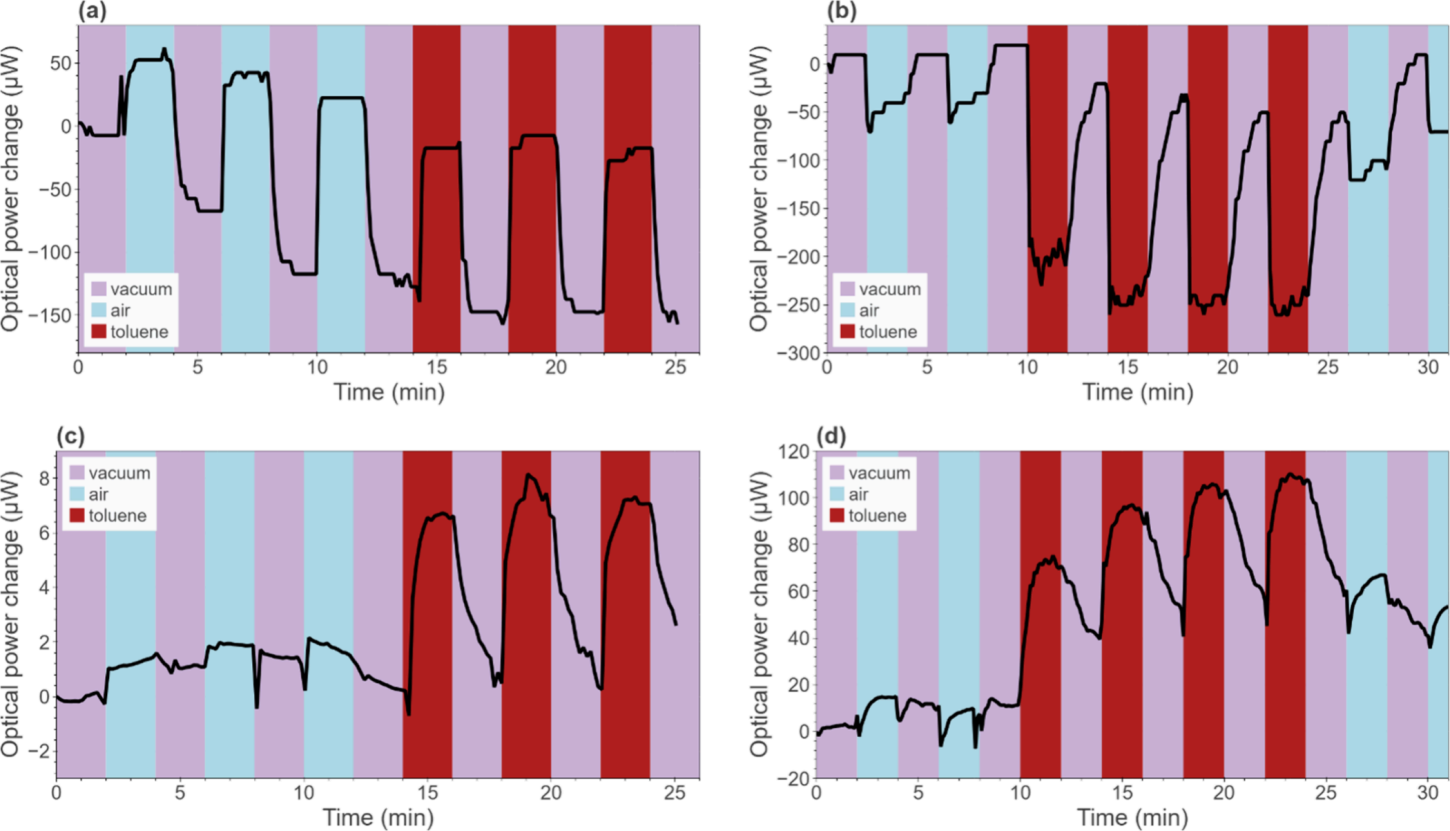
Optical power shifts observed in the (a, b) zeroth and (c, d) first orders of diffraction during cycles of exposure to vacuum and sample exposure to ambient air or vapor toluene. The plots in (a, c) depict the response of the grating with a 120 nm depth, while the plots in (b, d) were obtained with the grating with a 530 nm depth.

Figure 7 shows the responses of the gratings with 120 (panels a and c) and 530 nm (panels b and d) surface relief depths when exposed to toluene. Figure 7a,b presents data obtained from the detectors placed in the zeroth diffraction order, while Figure 7c,d depicts the signal in the first order of diffraction. Multiple cycles of vacuum–air and vacuum–analyte were performed to show the repeatability of the grating response. In every plot, power detection always starts when the sample was already under vacuum, and the distinct response of the sensor is visible within 2 min, after stopping the vacuum pump and opening the chamber to the ambient air. The moment of exposure of the sample to the VOC is easily distinguishable in the first order of diffraction. A significant growth of optical power is always a response to the interaction of PDMS with the analyte. Simultaneously, power in the zeroth order is decreasing. This is evidence that the response is due to the change in the diffractive properties of the grating and not some other effect. The energy equilibrium between the diffraction orders (zeroth and first orders) is maintained, as predicted by Raman–Nath theory. According to this theory, when the phase modulation of the grating grows, diffraction efficiencies follow a squared Bessel function of the first kind, as we described previously and as shown in Figure 2. For gratings with still relatively low heights of spatial profile (below 800 nm), the diffraction efficiency in the first order is always expected to increase, accompanied by a decrease in the zeroth order, as a result of the phase modulation rise due to swelling and/or a change in the refractive index. The change in optical power in the zeroth diffraction order often reaches 300 μW, with the largest vacuum–air change observed at 500 μW. However, the difference in power change during exposure to air and to the VOC is relatively low. In contrast, in the first diffraction order, the change does not exceed 120 μW (Figure 7d) due to the lower initial power, but the analyte uptake is clearly evident. For a comparison of all results, see Figure S2.

**Figure 8:**
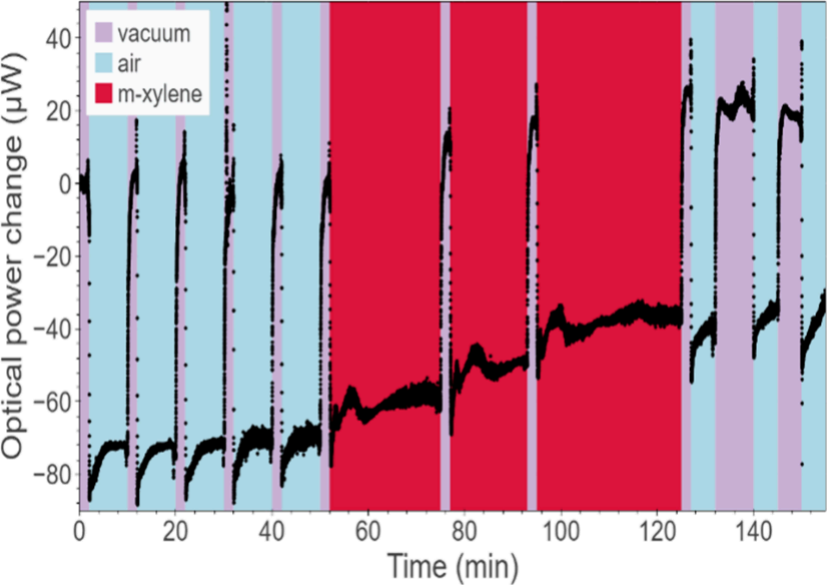
Cycles of evacuating and exposure to air and *m*-xylene of grating with a 530 nm deep surface profile, monitored in the first diffraction order. Concentration of *m*-xylene was 275 ppm.

The changes of power in the zeroth and first orders of diffraction when samples are exposed to air after degassing do not always mirror each other. Thus, these changes cannot be attributed to a change in the diffraction efficiency with certainty (for details, see the results in Figure S2). It is possible that this change is caused by a slight mechanical movement of the sample caused by the exposure to vacuum. Further investigations would be needed to confirm this hypothesis. It is worth noticing that the changes observed in the first diffraction order signal after exposure to air are small when compared to the changes after exposure to toluene, and this signal distinguishes between air and toluene much more reliably. The response of the gratings is noticeable immediately after exposure to the analyte following exposure to vacuum, and the optical power reaches a new value in around 20 s. However, during the first 10–20 s of response, also when the grating was exposed to air, a dynamic reaction of the sample response in the form of a sharp peak in the measured optical power was observed in some of the measurements (Figure 8). This most probably occurs because the gas is turbulently injected into the chamber due to the created underpressure and rapidly adsorbed into the PDMS. After that, the sensor reaches a state of equilibrium inside the chamber filled with a stationary gas. The sensor response is reversible, but a complete recovery to the state prior to the analyte uptake for some of the analytes requires some time. This is evident as the optical power decreases after opening the chamber to air again after vacuum but does not reach the initial state immediately.

The overall response of the sensor, especially in the context of response time, may be faster due to evacuating preceding the VOC insertion, as it removes air particles from the PDMS sample, making space for analyte molecules. Additionally, in some cases (mostly during the exposure to *m*-xylene and toluene) with subsequent cycles of evacuation and the introduction of the analyte, a gradual increase in optical power was observed. This may occur because the analyte is still deposited inside PDMS at the end of the evacuation cycle, and although the total amount of gas molecules in subsequent VOC uptakes may be the same, the final signal is higher. The same transducers (i.e., gratings listed in Table 3) were examined upon all five VOCs exposure to avoid added uncertainty from the analysis of different samples.

**Figure 9:**
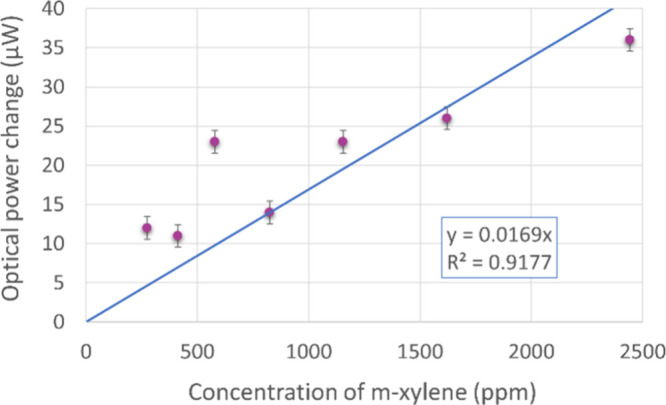
Optical response of grating with a 530 nm depth to varying concentrations of *m*-xylene. The linear fit was extrapolated to calculate the limit of detection. Error bars are the standard deviation estimated from mechanical instability during repeating vacuum–air cycles, which is 1.44 μW.

In the next part of the experiments, we investigated the response of the grating with the highest surface profile (530 nm) to varying concentrations of *m*-xylene and calculated the associated limit of detection (LOD) and sensing sensitivity from the linear regression line.^73^ By varying the quantity of liquid VOC injected into the gas development container, the sample was exposed to several concentrations, ranging from 275 to 2443 ppm (see the calculation method in the Supporting Information), as shown in Figure 9. Prior to each measurement, the sample was allowed to undergo evaporation of VOC molecules absorbed previously. Thus, we presume no or only a negligible amount of gas remains before each set of measurement cycles; however, further adsorption characterization would be necessary to deliver a comprehensive understanding of PDMS behavior in that regard. A complete stabilization of the optical power at a given value takes 5–20 min. For *m*-xylene and the surface grating with a 530 nm depth, the calculated sensitivity, i.e., the slope of the regression line, is 0.017 μW/ppm, while the estimated LOD is 186 ppm.

Figure 8 shows the results obtained for 275 ppm *m*-xylene, which was the lowest concentration examined experimentally. Responses to all *m*-xylene concentrations and the method of LOD calculation are summarized in Figure S3. The grating still exhibits a significant response at this concentration level, and the subsequent three exposures to the VOC show repeatable sensitivity after each *m*-xylene injection. Hence, three values for the change in the first order diffraction power can be clearly read, each with a higher baseline value compared to the previous response level, showing a stronger deposition of *m*-xylene inside the PDMS pores when the evacuation is applied over a short period of time. However, subsequent responses are predominantly lower (for the whole spectrum of concentrations examined), suggesting the proximity of the saturation point. Additionally, the overall amount of VOC molecules decreases with each cycle since gas is partially removed from the gas development container and redirected to the sample chamber, which is then evacuated. For the comparison shown in Figure 9 and the LOD calculation, only the first response to *m*-xylene in each measurement cycle was considered, which may be perceived as an ”empty/fresh” sensor response.

## Conclusions

In this study, we presented a new approach to employ PDMS surface relief gratings as an optical transducer for VOC detection. Taking advantage of the transparency and flexibility of PDMS, diffraction surface gratings with varying depths (120–530 nm) were successfully fabricated by copying sinusoidal master gratings inscribed holographically in acrylamide photopolymer. Each grating was exposed to five VOCs with different polarities and characterized by measuring their immediate response upon analyte uptake. The results revealed an increase in optical power diffracted into the first diffraction order when the gratings were exposed to VOCs. The increase in power in the first order of diffraction was consistently accompanied by a decrease in power in the zeroth diffraction order, thus confirming that a change of diffraction efficiency was taking place as a result of analyte uptake by the grating layer. Higher responses, reaching a 0.44% change in the diffraction efficiency, were observed for gratings with deeper surface profiles, which is consistent with the presented theoretical analysis. The investigated VOCs were toluene, *m*-xylene, chloroform, ethanol, and *n*-butanol, listed from those causing the highest to the lowest response. The obtained results were compared to the Hansen theory of solubility, proving a much stronger PDMS reaction to the nonpolar analytes. The estimated limit of detection and sensitivity for *m*-xylene are 186 ppm and 0.017 μW/ppm, respectively.

A significant advantage of the proposed approach is the ease of measuring optical power at a selected wavelength, as opposed to the spectroscopic wavelength shift observations performed in most optical fiber- and photonic crystal-based sensors. The potential device utilizing a PDMS diffractive structure can have a small footprint, is environmentally safe, and can be produced at a relatively low cost, thus justifying further work in improving the sensitivity and selectivity of these types of transducers.

Future sensor design involves the functionalization of surface relief PDMS gratings with dedicated sensing material(s), such as zeolite and metal–organic framework nanoparticles, both of which are known for their tunable VOC sorption properties. Upon surface coating with such materials, we anticipate significant enhancement in the sensitivity and selectivity of the transducer.

## Supporting Information Available

AFM images of surface profiles of PDMS relief gratings, responses of PDMS diffraction gratings as the optical power of light propagates in the zeroth and first diffraction orders, concentration calculation method, responses of the 530 nm deep grating to varying concentrations of *m*-xylene, and limit of detection calculation (PDF)
