# Supplementary material for: Optical Response of PDMS Surface Diffraction Gratings under Exposure to Volatile Organic Compounds
Source: ACS Appl Opt Mater. 2024 May 28;2(6):1188–97. doi: 10.1021/acsaom.4c00138 (PMC11220723; doi:10.1021/acsaom.4c00138)
Supplement: Supplementary file 1 — ot4c00138_si_001.pdf [file ot4c00138_si_001.pdf]

Supporting Information

Optical response of PDMS surface diffraction gratings under exposure to volatile organic compounds

Aleksandra Hernik, Faolan Radford McGovern, and Izabela Naydenova*

Centre for Industrial & Engineering Optics, School of Physics, Clinical & Optometric Sciences, Technological University Dublin, D07 ADY7 Dublin, Ireland

*izabela.naydenova@tudublin.ie

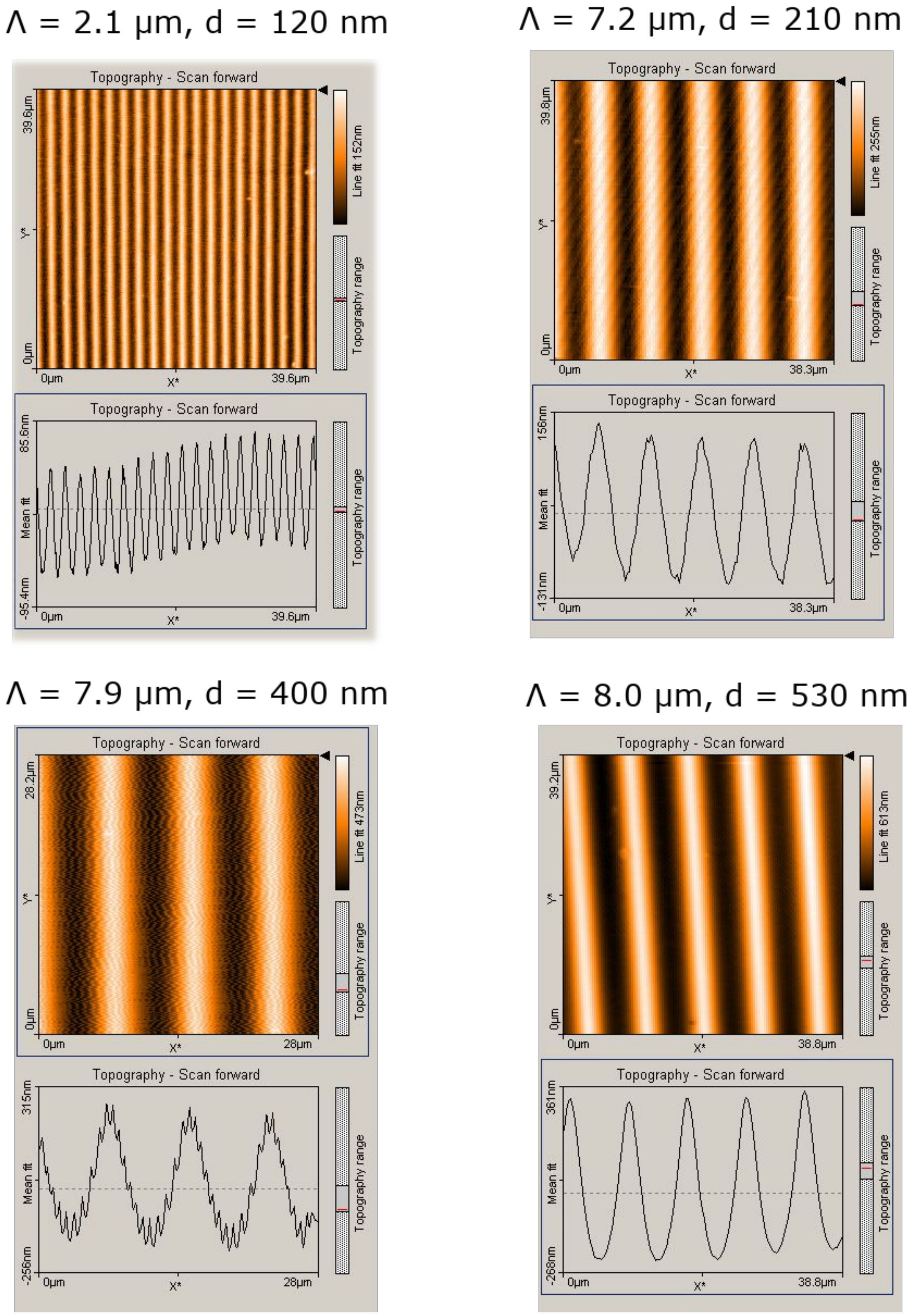

Fig. S1 *Atomic force microscopy images of surface profiles of PDMS relief gratings used in this study. Obtained with a Nanosurf Easyscan software. Λ is grating period, d is depth. Image of the grating with d = 490 nm and Λ = 8.0 µm was already depicted in Fig. 5.*

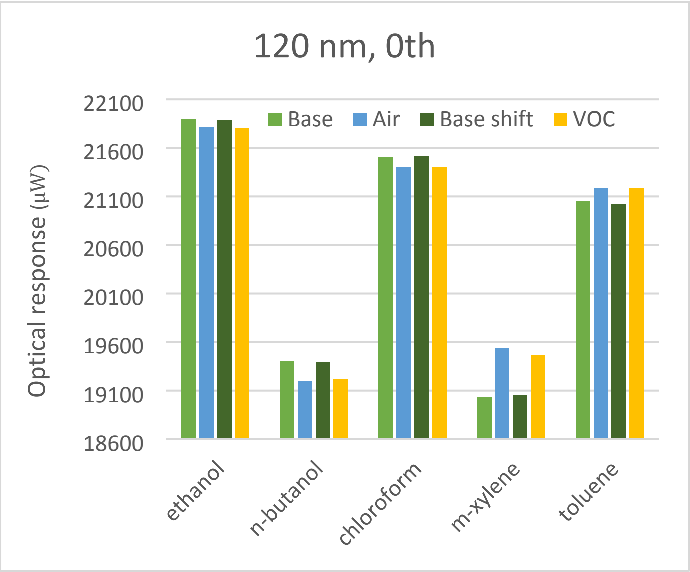
120 nm, 0th
Optical response (µW)
22100
21600
21100
20600
20100
19600
19100
18600
Base
Air
Base shift
VOC
ethanol
n-butanol
chloroform
m-xylene
toluene

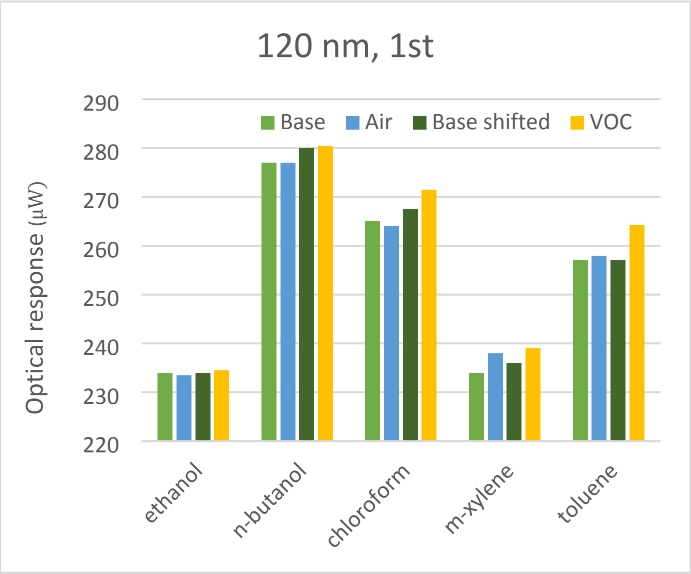
120 nm, 1st
Optical response (µW)
290
280
270
260
250
240
230
220
Base
Air
Base shifted
VOC
ethanol
n-butanol
chloroform
m-xylene
toluene

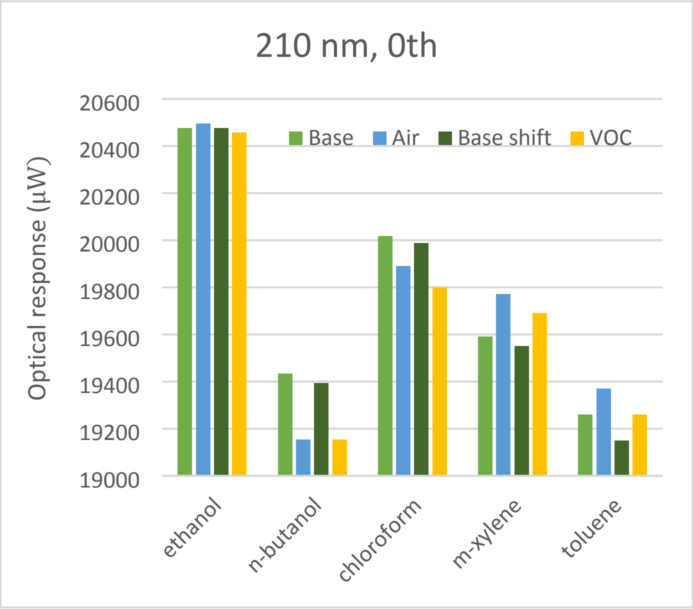
210 nm, 0th
Optical response (µW)
20600
20400
20200
20000
19800
19600
19400
19200
19000
Base
Air
Base shift
VOC
ethanol
n-butanol
chloroform
m-xylene
toluene

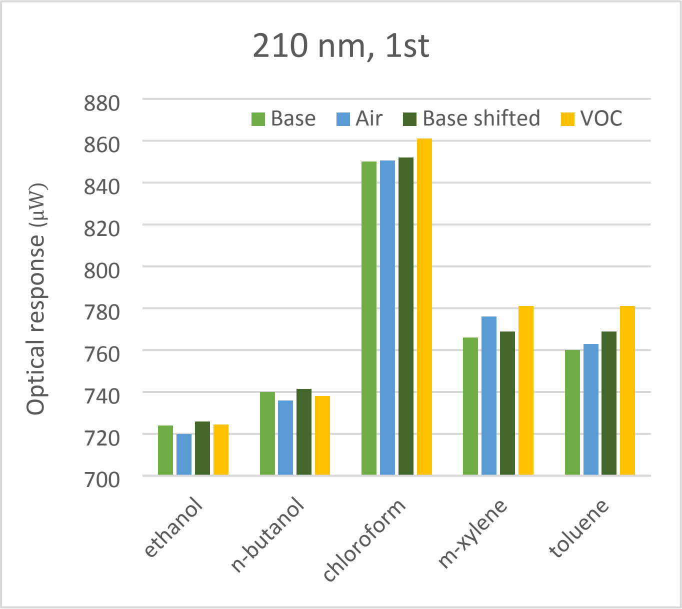
210 nm, 1st
Optical response (µW)
880
860
840
820
800
780
760
740
720
700
Base
Air
Base shifted
VOC
ethanol
n-butanol
chloroform
m-xylene
toluene

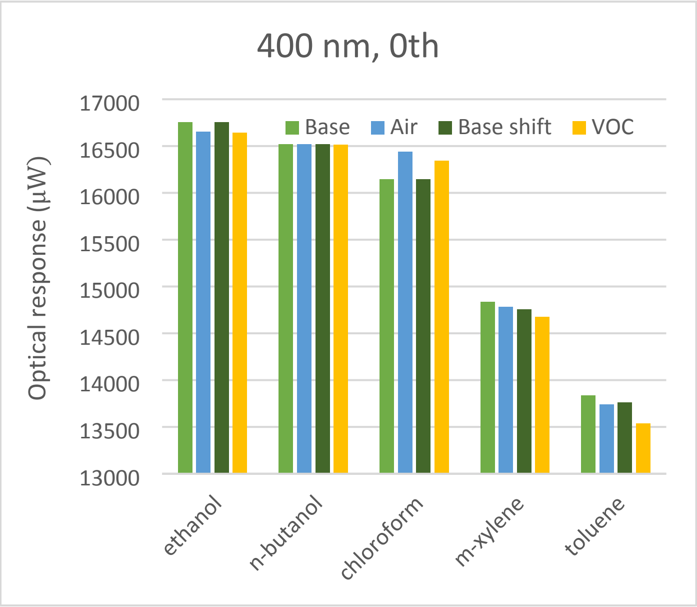
400 nm, 0th
Optical response (µW)
17000
16500
16000
15500
15000
14500
14000
13500
13000
Base
Air
Base shift
VOC
ethanol
n-butanol
chloroform
m-xylene
toluene

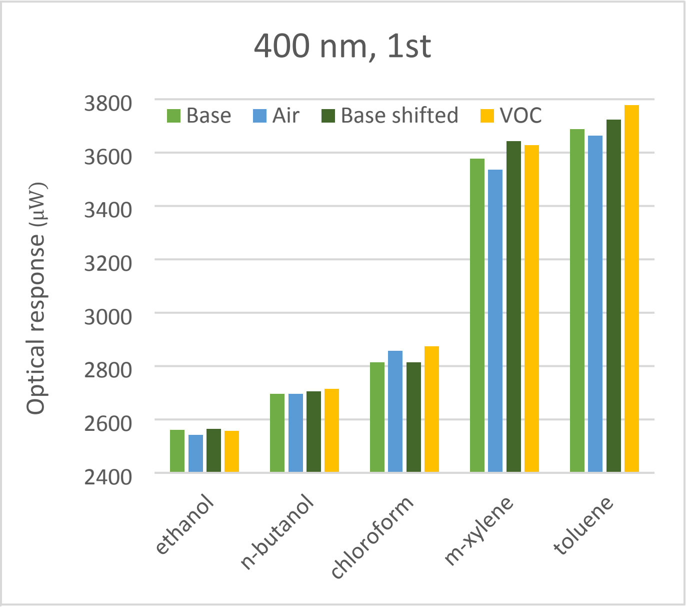
400 nm, 1st
Optical response (µW)
3800
3600
3400
3200
3000
2800
2600
2400
Base
Air
Base shifted
VOC
ethanol
n-butanol
chloroform
m-xylene
toluene

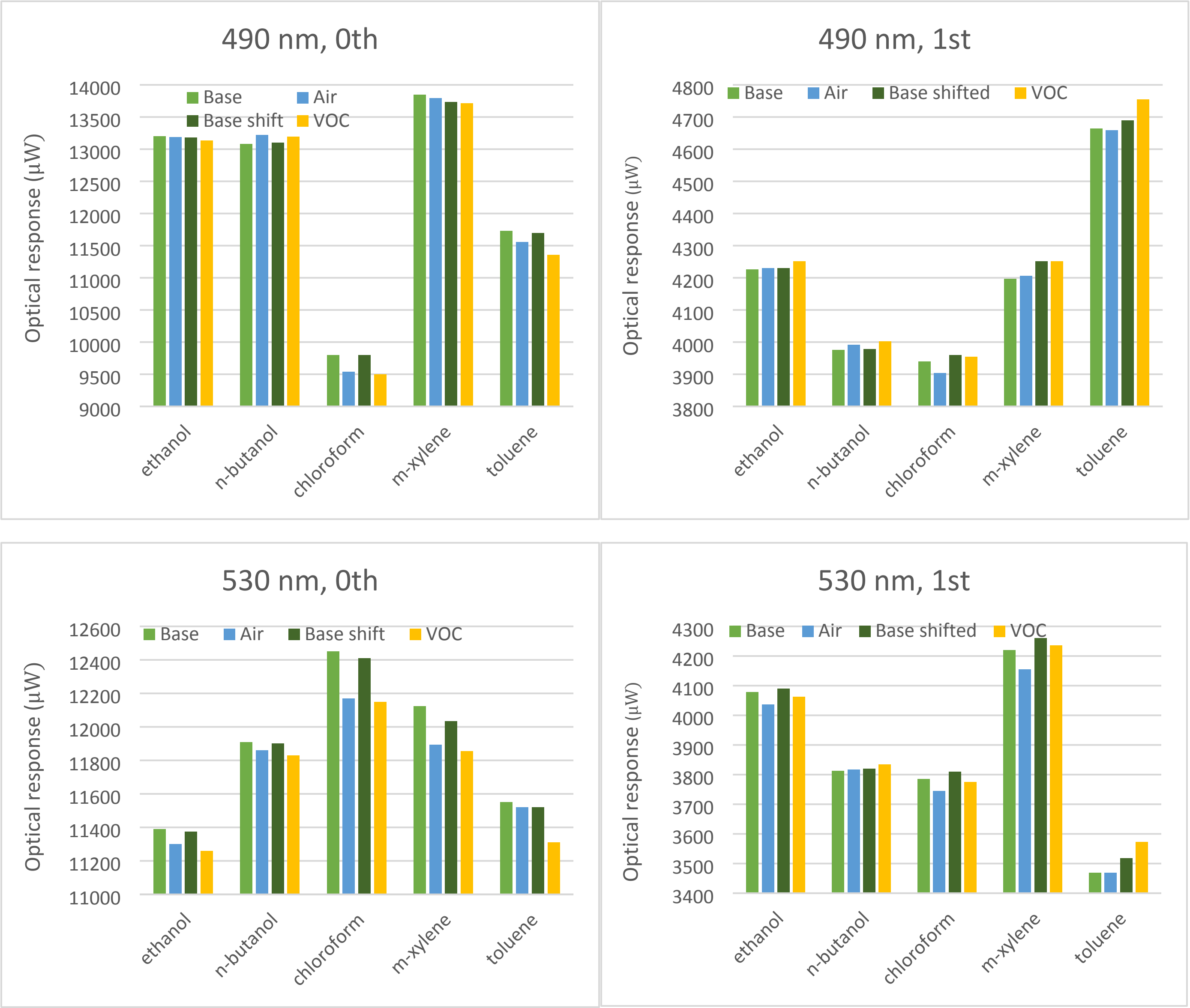

Fig. S2 *Summarised responses of the PDMS diffraction gratings with different depths as the optical power of light propagating in zeroth and first diffraction orders (as stated in the table's title). Base level – optical power measured when the air surrounding the grating was vacuuming out. Air – response to the ambient air. VOC – response after exposure to the selected VOC. Base shift – optical power during vacuuming but after VOC exposure. The sensing responses given in the Fig. 6 represent the difference between air and VOC level. There, an increase in the optical power in the first order is accompanied by a decrease of power in the zeroth order, indicating a change in the diffraction efficiency of the grating. During the initial step of vacuuming and exposure to air, the optical power shift is also visible but not correlated with sensing due to the same direction of power shift in both orders (mechanical interaction rather than power flow from zeroth to the first order). Different initial optical powers for each grating are present due to the slightly different spots on the grating illuminated during the gas exposure tests.*

Concentration calculations

From the ideal gas low, the number of moles of air in the gas development container: $n_{air} = \frac{pV}{RT}$

Where, at room conditions, p = 1 atm, V = 7.1 L, T = 294 K, R = 0.082 L atm k^{-1} mol^{-1}, thus n_{air} = 0.294.

Table S1. Properties of selected VOCs and the concentration generated in this study

VOC	density (g / ml)	M (g mol^-1)	Concentration (ppm) for 0.5 ml
toluene	0.86	92.1	15 646
m-xylene	0.86	106.2	13 572
chloroform	1.48	119.4	20 667
n-butanol	0.81	74.1	18 222
ethanol	0.79	46.1	28 265

Concentration (ppm): $C = \frac{n_{gas}}{n_{air}+n_{air}} = 10^6 \cdot \frac{V_{liq} \cdot density}{M} \frac{1}{n_{air} + \left(\frac{V_{liq} \cdot density}{M}\right)}$

Where V_{liq} is the amount of liquid injected into the gas development container. Here, V_{liq} = 0.5 ml

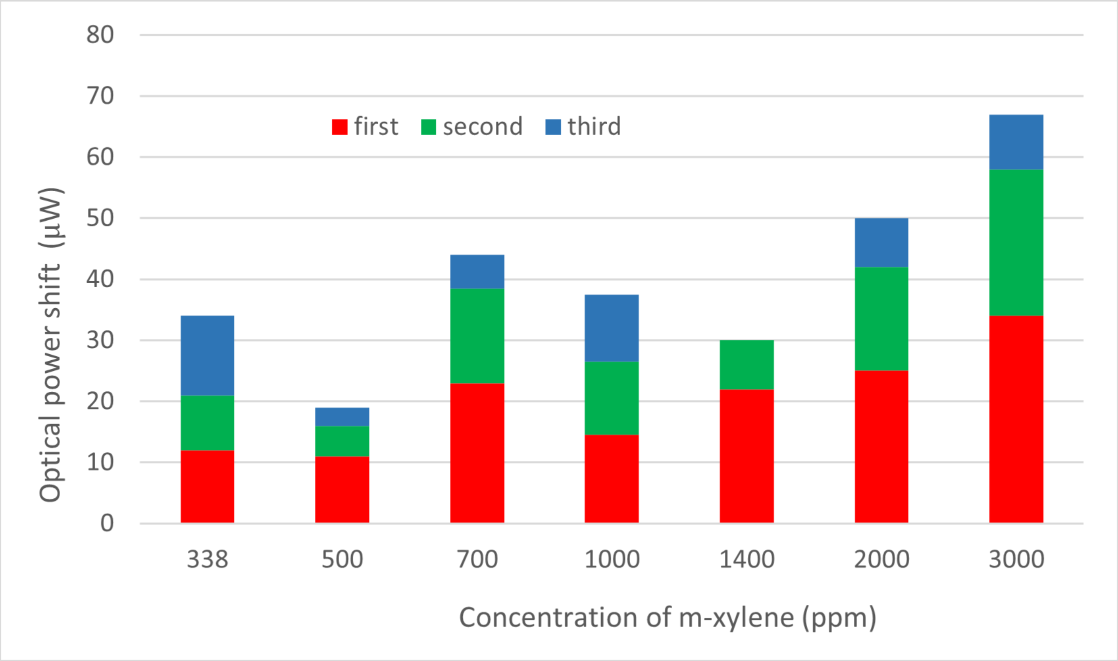

Fig. S3 *Summarised responses of the 530 nm deep grating to varying concentrations of m-xylene. Each column gathers three (two in case of 1400 ppm) subsequent responses to xylene with respect to the previous optical power level, i.e., the baseline increases with each VOC uptake inside the PDMS.*

The limit of detection was calculated from the calibration line at low concentrations:

$$LoD = \frac{3.3\ \sigma}{a}$$

Where a = 0.017 μW/ppm (sensitivity) is the slope of the line fitted to the data in Fig. 8. σ is the standard deviation of the response, estimated as 0.956 μW.

The above equation was chosen as the common method for the estimation of the detection limit, according to A. Shrivastava and V. Gupta (Methods for the Determination of Limit of Detection and Limit of Quantitation of the Analytical Methods. *Chronicles of Young Scientists* **2011**, 2 (1), 21).
